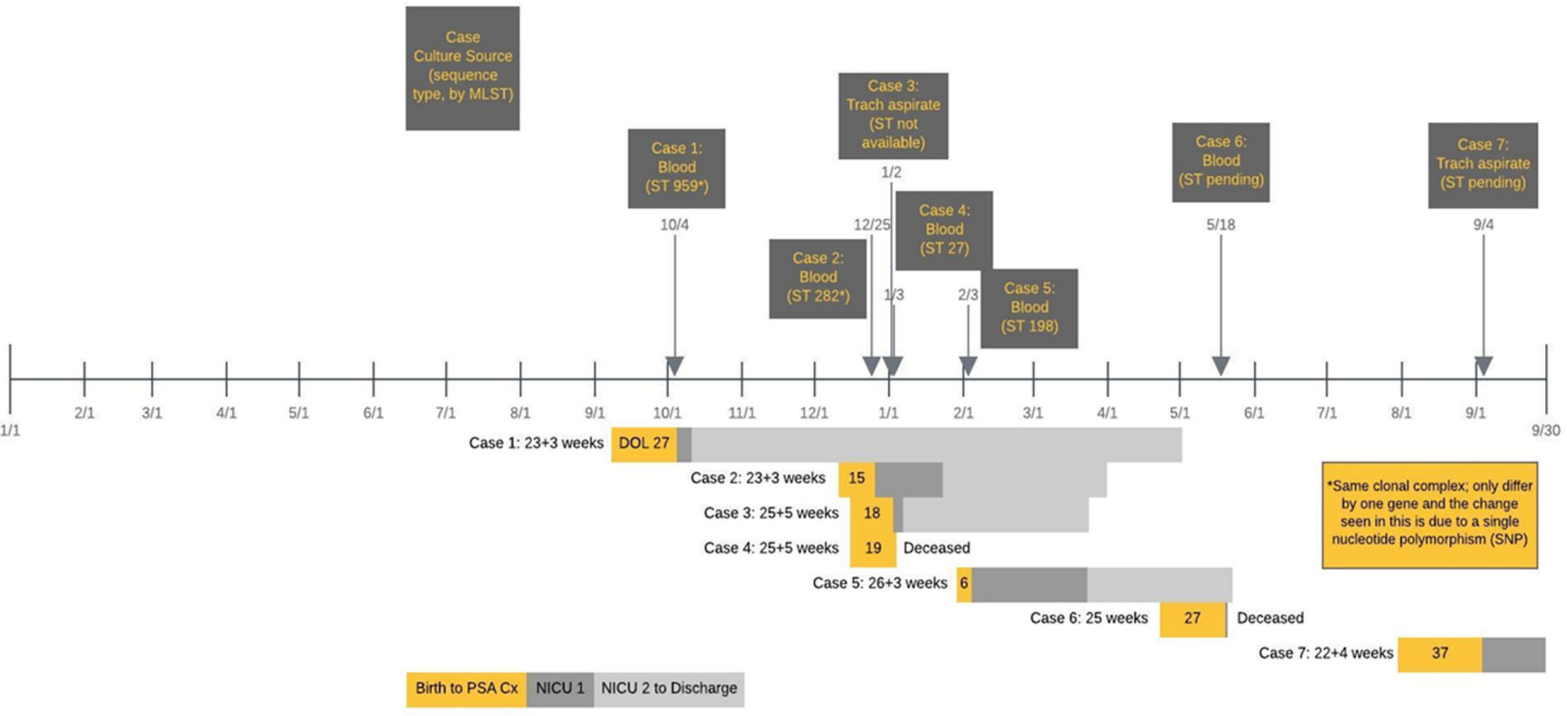# Cluster of Invasive *Pseudomonas aeruginosa* Infections in a Neonatal Intensive Care Unit

**DOI:** 10.1017/ash.2021.147

**Published:** 2021-07-29

**Authors:** Hillary Spencer, Ritu Banerjee, Gregory Wilson, Tanya Boswell

## Abstract

**Background:** *Pseudomonas aeruginosa* uncommonly causes illness in neonatal intensive care units (NICU). A cluster of 4 infections was appreciated over 6 weeks in our inborn–delivery NICU, prompting an investigation. **Methods:** Upon recognition of a cluster of infections, we retrospectively audited all cultures positive for *P. aeruginosa* from all sites (sterile and nonsterile) over the prior year in the index NICU (NICU 1, inborn) and for comparison in the adjacent NICU (NICU 2, larger, outborn–surgical). We performed multilocus sequence testing (MLST) of available clinical isolates to identify clonality. We initiated quarterly prospective surveillance of *P. aeruginosa* colonization of infants through nares, perirectal swabs, and tracheal aspirates of intubated infants or oropharyngeal swabs of nonintubated infants. We also swabbed incubators, ventilatory equipment, and sinks for selective *P. aeruginosa* culturing. **Results:** We identified 7 invasive *P. aeruginosa* infections in the inborn NICU (5 bloodstream and 2 pneumonia) over an 11-month period (Figure 1). Over the same period, there were no *P. aeruginosa* bloodstream infections in the adjacent NICU. Affected neonates were high risk: gestational age ranged from 22 weeks and 4 days to 26 weeks and 3 days; day of life at infection ranged from 6 to 37; 6 infants were on a jet ventilator; and all infants were receiving enteral nutrition (6 of 7 with donor human milk and 7 of 7 with expressed mother’s milk). Two infants died from their infection, and 5 infants survived to hospital discharge. All 7 isolates were pansusceptible to routine antimicrobials. MLST of the first 4 available isolates demonstrated 4 different sequence types; however, the first 2 were from the same clonal complex, indicating relatedness (Figure 1). For environmental samples, 8 obtained 8 cultures (swabs) of incubators and ventilatory equipment, and 24 cultures of faucets and drains of all sinks. Only sink cultures were positive, yielding 3 isolates identified as *P. aeruginosa* and 4 isolates identified as *P. aeruginosa–*like. Whole-genome sequencing (WGS) is underway to identify relatedness to the clinical isolates. We initiated quarterly infant surveillance by swab culture for *P. aeruginosa* nasal colonization then escalated to perirectal and oropharyngeal swab or tracheal aspirate cultures (intubated infants) in subsequent quarters. We did not detect any infants colonized with *P. aeruginosa*. **Conclusions:** We identified a cluster of *P. aeruginosa* in high-risk neonates with no point source. Molecular typing indicated a multiclonal cluster. This finding poses a management dilemma. A colonized water system is suspected and WGS of environmental samples is underway.

**Funding:** No

**Disclosures:** None

**Figure 1.**